# Major dietary patterns and risk of frailty in older adults: a prospective cohort study

**DOI:** 10.1186/s12916-014-0255-6

**Published:** 2015-01-20

**Authors:** Luz M León-Muñoz, Esther García-Esquinas, Esther López-García, José R Banegas, Fernando Rodríguez-Artalejo

**Affiliations:** CIBER of Epidemiology and Public Health (CIBERESP), Arzobispo Morcillo, s/n 28029, Madrid, Spain; Department of Preventive Medicine and Public Health, School of Medicine, Universidad Autónoma de Madrid/IdiPaz, Arzobispo Morcillo, s/n 28029, Madrid, Spain

**Keywords:** Cohort study, Diet, Frailty, Older adults, Spain

## Abstract

**Background:**

There is emerging evidence of the role of certain nutrients as risk factors for frailty. However, people eat food, rather than nutrients, and no previous study has examined the association between dietary patterns empirically derived from food consumption and the risk of frailty in older adults.

**Methods:**

This is a prospective cohort study of 1,872 non-institutionalized individuals aged ≥60 years recruited between 2008 and 2010. At baseline, food consumption was obtained with a validated diet history and, by using factor analysis, two dietary patterns were identified: a ‘prudent’ pattern, characterized by high intake of olive oil and vegetables, and a ‘Westernized’ pattern, with a high intake of refined bread, whole dairy products, and red and processed meat, as well as low consumption of fruit and vegetables. Participants were followed-up until 2012 to assess incident frailty, defined as at least three of the five Fried criteria (exhaustion, weakness, low physical activity, slow walking speed, and unintentional weight loss).

**Results:**

Over a 3.5-year follow-up, 96 cases of incident frailty were ascertained. The multivariate odds ratios (95% confidence interval) of frailty among those in the first (lowest), second, and third tertile of adherence to the prudent dietary pattern were 1, 0.64 (0.37–1.12), and 0.40 (0.2–0.81), respectively; *P*-trend = 0.009. The corresponding values for the Westernized pattern were 1, 1.53 (0.85–2.75), and 1.61 (0.85–3.03); *P*-trend = 0.14. Moreover, a greater adherence to the Westernized pattern was associated with an increasing risk of slow walking speed and weight loss.

**Conclusions:**

In older adults, a prudent dietary pattern showed an inverse dose-response relationship with the risk of frailty while a Westernized pattern had a direct relationship with some of their components. Clinical trials should test whether a prudent pattern is effective in preventing or delaying frailty.

## Background

Frailty is a medical syndrome resulting from age-associated impairments in several physiological systems. This syndrome is characterized by a high vulnerability to even minor environmental stressors (e.g., a minor infection), which leads to increased risk of disability, dependency, need for long-term care, and mortality [1]. Given the elevated prevalence of this syndrome [2,3] and its serious consequences, there is substantial interest in the identification of the risk factors for frailty, as well as in the development of interventions to avoid or delay its onset [4,5].

Emerging evidence suggests that low intake of certain micronutrients and protein could be a risk factor for frailty [6,7]. However, people do not eat nutrients; rather, they eat meals consisting of foods with complex combinations of nutrients that may interact. Moreover, intake of several nutrients is usually correlated (e.g., meat protein and saturated fat, vitamin A and C). Therefore, it might be difficult to assess the independent health effect of nutrients. Additionally, the effect of a single nutrient might be too small to be detected, but the joint effect of many nutrients within a dietary pattern could be large enough to be detectable. Accordingly, the investigation of dietary patterns can complement the study of individual nutrients and overcome its potential limitations [8].

Two main approaches have been used to describe dietary patterns [8,9]. The first approach formulates indices or scales representing the degree of adherence to *a priori*-defined dietary patterns that are deemed to be healthy according to the prevailing scientific evidence. A good example frequently used in the literature is the Trichopoulou index, which represents the traditional Mediterranean diet [10]. The second approach uses factor or cluster analysis to empirically define a few food patterns that reflect different dietary compositions. These patterns derived *a posteriori* have the advantage of reflecting existing food habits in the study population. These two approaches address different research questions. For instance, the traditional Mediterranean diet, defined *a priori*, represents the diet consumed by populations in the Mediterranean basin during the 1960s, whereas a pattern defined *a posteriori* represents the present-day dietary intake. This is important because, in recent decades, diet in Mediterranean countries has undergone a process of westernization, which includes a large increase in the consumption of red meat, saturated fats and simple carbohydrates, and reduced consumption of whole-grain cereals and legumes [11].

In a recent paper using information from the Seniors-ENRICA cohort, we have found that adherence to the Mediterranean diet, as assessed by *a priori* scales including the Trichopoulou index, was associated with a lower risk of frailty [12]. Further, in previous research, an index of global diet quality [13] and several *a priori* scales of Mediterranean diet adherence [14-17] have been linked to lower frequency of frailty or some of its components. Herein, we have performed a factor analysis with food consumption data from the Seniors-ENRICA cohort to identify existing dietary patterns in the population. To our knowledge, no previous study has yet examined the association between *a posteriori*-defined dietary patterns and the risk of frailty in older adults.

## Methods

### Study design and population

Data were taken from the Seniors-ENRICA cohort, whose methods have been previously reported [12,18]. In brief, this cohort was established between 2008 and 2010 with 2,614 non-institutionalized individuals aged 60 years and older. At baseline, information was collected by telephone interview and by a face-to-face questionnaire and a physical examination conducted at the participants’ homes. Participants were followed-up until 2012, when a second wave of data collection was performed. Ninety-five participants (3.6%) died during follow-up; from the remaining 2,519 subjects, 2,085 had complete information on frailty in 2012. Baseline socio-demographic, lifestyle, and clinical characteristics of individuals lost to follow-up and those remaining in the study were similar, although the latter were slightly younger and had higher educational level and less comorbidity.

Study participants gave written informed consent. The study was approved by the Clinical Research Ethics Committee of the University Hospital La Paz in Madrid.

### Study variables

#### Dietary patterns derived from food consumption data

At baseline, food consumption was assessed with a validated computerized dietary history which was developed from that used in the EPIC-cohort study in Spain [19,20]. This instrument registered the consumption of 880 foods in the preceding year, and quantification of food portions was aided by a set of photographs.

To identify dietary patterns, the 880 foods were categorized into 36 different groups according to similarities in nutrient profile. We applied factor analysis (principal components analysis) to these food groups to generate various independent dietary patterns (factors) made up of foods with a high degree of inter-correlation [21]. The factors were rotated by orthogonal transformation (Varimax rotation) to facilitate their interpretation. Dietary patterns to be retained for future analysis took into account their ease of interpretation, and required an eigenvalue of ≥1.5 on the scree test [22]. Factor loadings were obtained for each food group to identify the groups most closely correlated with the dietary pattern (Table 1). Two patterns were identified. The first was called the ‘prudent’ pattern (PP) due to the high consumption of olive oil, vegetables, potatoes, legumes, blue fish, pasta, and meat; and the second was called the ‘Westernized’ pattern (WP) because of the high consumption of refined bread, whole dairy products, and red and processed meat, as well as the low intake of whole grains, fruit, low-fat dairy, and vegetables. For each pattern, each subject received a score that was calculated as the sum of the intakes in each food group weighted by the corresponding factor loading. A higher score indicated a higher adherence to the respective dietary pattern.

**Table 1:** **Factor-loading matrix for dietary patterns among the non-frail population at baseline**

	**Prudent pattern**	**Westernized pattern**
**Foods**		
Olive oil	0.726	*
Non-leafy vegetables	0.608	−0.303
Leafy vegetables	0.539	−0.228
Potatoes (other than French fries) and other tuber products	0.418	*
Legumes (non-soy-derived products)	0.394	*
Blue fish	0.341	*
Pasta	0.314	0.185
Sauces	0.313	0.200
Poultry and other unprocessed white meat	0.280	−0.163
Refined grains and breakfast cereals	0.268	*
Non-blue fish and seafood	0.266	−0.152
Eggs	0.214	0.194
Nuts and oily seeds	0.184	*
Water, infusions, non-sweetened non-alcoholic beverages	*	*
Preserved fruits and olives	*	*
Refined bread products	0.264	0.543
Whole dairy products	*	0.397
French fries	0.247	0.382
Red and processed meat	0.246	0.379
Sugar and honey	*	0.334
Beer, wine and cider	*	0.304
Pastries	*	0.299
Non-olive oils	*	0.284
Sweetened non-alcoholic beverages	*	0.278
Hard liquor (whisky, gin, rum, brandy, vodka)	*	0.222
Appetizers	*	0.174
Biscuits	*	*
Butter, margarine and lard	*	*
Organ meats	*	*
Coffee and tea	*	*
Chocolate and cocoa	*	*
Other sweets (jam, jelly, marzipan, candies) and sweetener	*	*
Soy-derived products	*	*
Low-fat dairy products	*	−0.363
Fresh fruit and fruit juices	*	−0.391
Whole grain products	*	−0.430

*Correlation coefficient between 0.15 and −0.15; n = 1,872.

#### Frailty

At baseline and at the end of follow-up, we used a slight modification of the operational definition of frailty developed in the Cardiovascular Health Study by Fried et al. [23]. Specifically, frailty was defined as having three or more of the following five criteria: i) Exhaustion*:* any of the following responses to two questions taken from the CES-D scale [24]: “I felt that everything I did was an effort” and “I could not get going” at least 3 to 4 days a week. ii) Weakness: the lowest quintile in our study sample for maximum strength on the dominant hand adjusted for sex and body mass index (BMI). Strength was measured as the highest of two consecutive measures with a Jamar dynamometer [25,26]. iii) Low physical activity: lowest quintile in our sample (walking ≤2.5 h/week in men and ≤2 h/week in women). iv) Slow walking speed: the lowest quintile in our study sample for the three-meter walking speed test, adjusted for sex and height [26,27]. v) Weight loss: involuntary loss of ≥4.5 kg of body weight in the preceding year.

#### Potential confounders

At baseline, information was gathered on socio-demographic variables, lifestyle, and diseases that could act as confounders of the study association because of their relation to both diet and frailty. Specifically, study participants were asked about their sex, age, educational level, the last occupation held, tobacco consumption, number of medications used, and energy intake (calculated with standard food composition tables). Occupation was coded according to the National Classification of Occupations in Spain, and was classified into four classes: I (professionals, managers, proprietors, and clerical workers); II (self-employed farm workers); III (skilled and unskilled manual workers); and IV (paid farm workers). Housewives were assigned the occupation of their husband. Study participants also reported the following physician-diagnosed diseases: cardiovascular disease (myocardial infarction, stroke, heart failure), diabetes mellitus, cancer at any site, asthma or chronic bronchitis, osteomuscular disease (osteoarthritis, arthritis, hip fracture), and depression requiring drug treatment. Weight and height were measured in standardized conditions [28] and the BMI was calculated as weight in kg divided by square of height in m. Lastly, cognitive function was assessed with the Mini-Mental State Examination [29] and limitations in instrumental activities of daily living (IADL) were ascertained with the Lawton-Brody questionnaire [30].

### Statistical analysis

From the 2,085 individuals who were followed-up, we excluded 174 who were frail or lacked data on frailty at baseline, 7 with missing data on diet, and 32 without data on potential confounders. Therefore, the analyses were conducted on 1,872 individuals.

The association between dietary patterns and risk of frailty was summarized with odds ratios (OR) and their 95% confidence interval (CI) obtained from logistic regression. Two logistic models were built: the first one adjusted for the baseline number of frailty criteria, sex, age, education, and occupation, and the second model additionally adjusted for the rest of the variables described above. Adherence to empirically-derived dietary patterns was classified into tertiles, and the lowest tertile was used as the reference group. *P* values for linear trend were calculated by modeling tertiles of the dietary patterns as a continuous variable.

We ran several sensitivity analyses to assess the robustness of the main results. Given that the study aimed to evaluate the association between diet and frailty, we repeated the analysis excluding the weight loss criterion from the definition of frailty [14]. Thus, in this analysis frailty was defined as having at least two of the four remaining Fried criteria. Also, we replicated the analyses after excluding the following individuals: i) those reporting severe or substantial difficulty in chewing or eating at the end of follow-up; ii) those with diagnosed disease at baseline (cardiovascular disease, diabetes, cancer, chronic lung disease, or depression requiring treatment); and iii) those with baseline limitation in IADL.

Finally, we used the same type of modeling to assess the association between dietary patterns and the risk of each frailty criterion among robust individuals (free of all five of Fried’s criteria) at baseline.

Statistical significance was set at two-sided *P* <0.05. The analyses were performed with Stata®, version 11.1.

## Results

Compared to individuals in the first (lowest) tertile of the PP, those in the third tertile were younger, had a higher energy intake, and there was a lower percentage of smokers, persons diagnosed with diabetes or depression, and with limitations in IADL; however, there was a higher percentage of individuals with BMI ≥30 kg/m^2^ and reporting osteomuscular disease. However, it should be noted that for depression, IADL limitation, and BMI, the data did not suggest a linear association with the PP. As regards the WP, those in the third tertile were younger and showed a higher energy intake, there was a greater percentage of smokers and individuals with limitation in IADL, and a lower percentage of subjects with university education and osteomuscular disease (Table 2). Nevertheless, for IADL limitation and university education, we found no indication of a linear association with the WP.

**Table 2:** **Characteristics of the non-frail population at baseline, by adherence to the dietary patterns**

	**Prudent pattern**				**Westernized pattern**			
	**Tertile 1**	**Tertile 2**	**Tertile 3**	***P*** **value**	**Tertile 1**	**Tertile 2**	**Tertile 3**	***P*** **value**
n	620	627	625		624	625	623	
Number of frailty components, mean (SE)	0.3 (0.1)	0.3 (0.1)	0.2 (0.1)	<0.01	0.2 (0.1)	0.3 (0.1)	0.3 (0.1)	<0.01
Sex, women , %	51.1	51.8	51.8	0.53	51.6	51.5	51.5	0.55
Age, years, mean (SE)	70.1 (0.3)	68.4 (0.3)	67.6 (0.3)	<0.01	69.2 (0.3)	68.7 (0.3)	68.2 (0.3)	0.02
Educational level, %								
≤Primary	56.7	48.4	56.4	<0.01	51.7	56.3	53.6	<0.01
Secondary	22.9	27.4	24.8		24.2	25.2	25.7	
University	20.4	24.2	18.8		24.2	18.5	20.7	
Occupational social class, %								
I (Professionals, managers, proprietors and clerical workers)	61.9	66.0	63.0	0.04	66.7	60.5	63.7	0.07
II (Self-employed farm workers)	2.1	2.7	4.4		2.7	3.4	3.1	
III (Skilled and unskilled manual workers)	34.5	30.2	31.5		29.7	35.0	31.4	
IV (Paid farm workers)	1.5	1.1	1.1		0.9	1.1	1.8	
Tobacco, %								
Never smoker	61.6	59.1	60.3	<0.01	63.9	60.9	56.1	<0.01
Former smoker	25.5	29.4	30.4		29.6	26.4	29.2	
Current smoker	12.9	11.5	9.3		6.5	12.7	14.6	
Body mass index, kg/m^2^, %								
<25	19.5	18.6	20.1	0.002	20.8	18.8	18.5	0.01
25–29.9	49.6	53.4	46.5		49.6	50.6	49.4	
≥30	30.8	28.0	33.4		29.6	30.5	32.1	
Energy intake, kcal/d, mean (SE)	1731 (18)	2009 (18)	2358 (18)	<0.01	1854 (19)	1922 (19)	2324 (19)	<0.01
Mini-Mental State Examination, mean (SE)	27.8 (0.1)	28.2 (0.1)	28.2 (0.1)	<0.01	28.2 (0.1)	28.1 (0.1)	27.9 (0.1)	<0.01
Eating difficulties, %	3.3	2.9	2.6	0.11	2.5	2.3	4.0	0.03
Diagnosed diseases, %								
Cardiovascular disease^a^	4.9	4.4	5.1	0.005	5.7	4.2	4.5	0.002
Diabetes	13.0	11.9	9.7	<0.01	12.2	10.1	12.2	<0.01
Cancer	1.2	2.1	2.2	0.19	2.0	1.8	1.7	0.42
Asthma or chronic bronchitis	7.5	7.8	7.1	0.15	7.5	8.2	6.8	0.12
Osteomuscular disease^b^	43.5	47.6	49.8	<0.01	48.1	47.9	44.9	<0.01
Depression needing treatment	7.4	4.6	4.8	<0.01	5.2	5.3	6.2	<0.01
Limitation in IADL, %	9.0	6.0	7.5	<0.01	6.8	6.7	8.9	<0.01
Number of treatments, mean (SE)	2.1 (0.1)	1.9 (0.1)	1.9 (0.1)	<0.01	2.2 (0.1)	1.8 (0.1)	1.8 (0.1)	<0.01

SE, Standard error; IADL, Instrumental activities of daily living. Values are adjusted for sex and age. *P* values in Table 2 test differences between the three tertile groups, based on χ^2^ or ANOVA tests, as appropriate.

^a^Ischemic heart disease, stroke, and heart failure; ^b^Osteoarthritis, arthritis, and hip fracture; n = 1,872.

During a mean follow-up of 3.5 years, we ascertained 96 cases of incident frailty. Table 3 shows the association between adherence to the dietary patterns and the risk of frailty. Results from models with partial (model 1) and full (model 2) adjustment were rather similar, and showed that the PP was inversely associated with the risk of frailty while the WP had a non-statistically significant tendency to a higher risk. In model 2, the OR (95% CI) of frailty among those in the first, second, and third tertile of adherence to the PP were 1, 0.64 (0.37–1.12), and 0.40 (0.2–0.81), respectively; *P*-trend = 0.009. The corresponding values for the WP were 1, 1.53 (0.85–2.75), and 1.61 (0.85–3.03); *P*-trend = 0.14. For both patterns, these results were similar to those obtained in all the sensitivity analyses.

**Table 3:** **Association between dietary patterns and risk of frailty during a 3.5-year follow-up of older adults**

	**Prudent pattern**				**Westernized pattern**			
	**Tertile 1**	**Tertile 2**	**Tertile 3**		**Tertile 1**	**Tertile 2**	**Tertile 3**	
		**Odds ratio**	**Odds ratio**	***P-*** **trend**		**Odds ratio**	**Odds ratio**	***P*** **-trend**
		**(95% CI)**	**(95%** **CI)**			**(95% CI)**	**(95% CI)**	
**Main analysis**								
Number of frailty events	48	27	21		24	35	37	
Model 1	Ref.	0.69 (0.41–1.17)	0.59 (0.33–1.04)	0.05	Ref.	1.53 (0.87–2.72)	1.61 (0.91–2.84)	0.11
Model 2	Ref.	0.64 (0.37–1.12)	0.40 (0.20–0.81)*	0.009	Ref.	1.53 (0.85–2.75)	1.61 (0.85–3.03)	0.14
**Sensitivity analyses**								
*Excluding weight loss from the definition of frailty*								
Number of frailty events	102	78	62		71	81	90	
Model 1	Ref.	0.80 (0.57–1.13)	0.72 (0.50–1.03)	0.07	Ref.	1.23 (0.86–1.76)	1.43 (1.00–2.03)*	0.04
Model 2	Ref.	0.83 (0.58–1.18)	0.67 (0.44–1.02)	0.06	Ref.	1.27 (0.88–1.83)	1.53 (1.03–2.26)*	0.03
*Excluding 58 individuals with eating difficulty*								
Number of frailty events	40	23	13		20	31	31	
Model 1	Ref.	0.70 (0.40–1.23)	0.63 (0.34–1.15)	0.11	Ref.	1.69 (0.92–3.13)	1.61 (0.87–2.98)	0.14
Model 2	Ref.	0.64 (0.35–1.18)	0.40 (0.19–0.84)*	0.01	Ref.	1.64 (0.87–3.11)	1.45 (0.73–2.90)	0.28
*Excluding 543 individuals with diagnosed severe diseases*								
Number of frailty events	29	12	10		14	19	18	
Model 1	Ref.	0.54 (0.26–1.13)	0.48 (0.22–1.05)	0.05	Ref.	1.30 (0.61–2.76)	1.34 (0.62–2.87)	0.46
Model 2^a^	Ref.	0.52 (0.24–1.16)	0.34 (0.13–0.89)*	0.02	Ref.	1.23 (0.57–2.67)	1.44 (0.63–3.29)	0.38
*Excluding 182 individuals with limitation in IADL* ^*b*^								
Number of frailty events	32	17	10		15	22	22	
Model 1	Ref.	0.55 (0.29–1.03)	0.40 (0.19–0.85)*	0.01	Ref.	1.56 (0.78–3.10)	1.39 (0.69–2.80)	0.37
Model 2	Ref.	0.49 (0.25–0.98)*	0.26 (0.11–0.63)**	0.002	Ref.	1.70 (0.84–3.44)	1.36 (0.63–2.94)	0.40

CI, Confidence interval; IADL, Instrumental activities of daily living; *P <0.05, **P <0.01.

Model 1: Adjusted for number of frailty components at baseline (0, 1, 2), sex, age, educational level (≤primary, secondary, university), and occupation (social class I, II, III, IV).

Model 2: Adjusted additionally for tobacco (never smoker, former smoker, current smoker), body mass index (kg/m^2^), energy intake (kcal/d), cardiovascular disease, diabetes mellitus, cancer, asthma or chronic bronchitis, osteomuscular disease, depression requiring treatment, number of drug treatments, and score on the Mini-Mental State Examination.

^a^As model 2 above but without adjustment for cardiovascular disease, diabetes mellitus, cancer, asthma or chronic bronchitis, and depression requiring treatment; ^b^Another 28 individuals had missing data on IADL; n = 1,872.

Finally, a greater adherence to the PP showed a non-statistically significant tendency to a lower risk of exhaustion and of slow walking speed (Table 4). However, the WP did show an association with an increasing risk of slow walking speed and weight loss. Specifically, the OR (95% CI) of slow walking speed across tertiles of the WP were 1, 1.15 (0.74–1.76), and 1.85 (1.19–2.87); *P*-trend = 0.007. For weight loss, the corresponding values were 1, 1.37 (0.77–2.41), and 2.12 (1.22–3.70); *P*-trend = 0.007. Results for the association between the WP and exhaustion and low physical activity were in the same direction but did not reach statistical significance (Table 4).

**Table 4:** **Association between dietary patterns and risk of each frailty criterion during a 3.5-year follow-up of robust older adults at baseline**

	**Prudent pattern**				**Westernized pattern**			
	**Tertile 1**	**Tertile 2**	**Tertile 3**	***P*** **-trend**	**Tertile 1**	**Tertile 2**	**Tertile 3**	***P-*** **trend**
**Frailty criteria**								
**Exhaustion,** n events	57	42	39		40	50	48	
Odds ratio (95% CI)	Ref.	0.83 (0.53–1.30)	0.75 (0.44–1.26)	0.27	Ref.	1.40 (0.89–2.21)	1.58 (0.96–2.58)	0.07
**Low physical activity,** n events	53	80	68		52	70	79	
Odds ratio (95% CI)	Ref.	1.46 (0.99–2.17)	0.95 (0.61–1.50)	0.77	Ref.	1.38 (0.98–2.04)	1.46 (0.96–2.21)	0.08
**Slow walking speed,** n events	71	64	56		56	55	80	
Odds ratio (95% CI)	Ref.	0.83 (0.55–1.25)	0.74 (0.46–1.18)	0.20	Ref.	1.15 (0.74–1.76)	1.85 (1.19–2.87)*	0.007
**Weight loss,** n events	34	29	40		24	30	49	
Odds ratio (95% CI)	Ref.	0.76 (0.44–1.30)	0.80 (0.46–1.41)	0.46	Ref.	1.37 (0.77–2.41)	2.12 (1.22–3.70)*	0.007
**Muscle weakness,** n events	95	75	68		90	77	71	
Odds ratio (95% CI)	Ref.	0.93 (0.64–1.35)	0.95 (0.62–1.46)	0.81	Ref.	1.01 (0.70–1.45)	1.09 (0.73–1.63)	0.68

CI, Confidence interval; *P <0.01.

Analyses adjusted as in model 2 in Table 3; n = 1,486.

## Discussion

Our results show that adherence to a PP, characterized by high intake of olive oil and vegetables, had an inverse dose-response relationship with frailty; in contrast, an increasing adherence to the WP, characterized by high intake of refined cereals, whole dairy, and red and processed meat, was associated with increased the risk of slow walking speed and weight loss.

Previous population-based surveys and cohort studies with selected samples have found dietary patterns consistent with the PP, which has also been called the ‘healthy’ or ‘whole food’ pattern, and with the WP, also called the ‘processed food’ pattern. As in our study, the PP has usually been associated with a healthier lifestyle, while the WP has been linked to less healthy behaviors [31,32].

In cohort studies, the PP has been associated with lower risk of coronary disease [31,33] and diabetes [34]. It has also shown an inverse cross-sectional association with the metabolic syndrome and insulin resistance [35], as well as with levels of numerous biomarkers of inflammation and cardiovascular risk [32,36]. Moreover, some studies have found the PP to be associated with lower risk of cognitive impairment [37,38] and depression [39,40]; however, a cross-sectional study found no association with cognitive function [41] and in one prospective investigation the protection against depression, was restricted to individuals older than 60 years [42]. As regards the WP, it has been linked to higher risk of coronary disease [31,33], diabetes [34], metabolic syndrome, and insulin resistance [35]. There is also evidence that the WP is associated with higher levels of cardiometabolic biomarkers, including insulin, C-peptide, leptin, homocysteine, and inflammation mediators (sTNFR2, IL-6, CRP, E-selectin) [34,36,43]. Finally, the WP has been related to an increased risk of poor cognition [37,41], while some [39], but not all studies [40,42], have reported a higher risk of depression. Given that obesity [44], systemic inflammation [45-47], cardiovascular disease [2], poor cognitive function [48,49], and depression [50] are all well-known risk factors of frailty, all these health disorders could contribute to the association between the PP, the WP, and frailty.

There is evidence that healthy diets, which are consistent with the PP in our study, may reduce the risk of several components of frailty. Specifically, a diet rich in vegetables, whole grains, and blue fish has been associated with higher grip strength in older adults [51]. Further, a healthy diet has been shown to protect against slow walking speed [13-17], unintentional weight loss [14,15], low physical activity [13,14], and muscle weakness [6]. Despite the clear inverse association between the PP and frailty in our study, the PP did not evidence a statistically significant association with any of its components, though it showed some tendency to reduce the risk of exhaustion and slow walking speed. This suggests that the protective effect of the PP on frailty might result from synergic benefits on each component of frailty, which are nevertheless too small to be detectable when assessed separately. We are not aware of previous investigations on the effect of the WP on frailty or its components; in our study, this pattern was directly associated with several frailty components, in particular slow walking speed and weight loss.

Although the Mediterranean diet, as assessed by *a priori* scales such as the Trichopoulou index [10,12], and the PP empirically identified in this study share a high consumption of olive oil and vegetables, there are substantial differences between these two dietary patterns. Specifically, some foods which are typical of the Mediterranean diet are not part of the PP. This is the case for fruits and alcoholic beverages (e.g., wine). The reverse situation is also true, so that consumption of white and red meat contributes to the PP, while consumption of any type of meat scores negatively in the Trichopoulou index; moreover, consumption of potatoes is a component of the PP but not of the traditional Mediterranean diet. As a result, the correlation between the PP and the Trichopoulou index was modest (Pearson r = 0.37) in our study sample.

Our results are unique in showing that, despite changes in the traditional diet in Spain derived from the socioeconomic development and modern living arrangements during the last decades [11], a PP characterized by intake of olive oil and vegetables, and also potatoes and meat, protects from the frailty syndrome in older adults. Of particular note is that the PP does not include alcoholic beverages; this is important because, in some studies, alcohol intake has been identified as one of the main contributors to the health benefits of the Mediterranean diet [52,53]. Given that older adults frequently consume alcohol-interacting medications and are particularly vulnerable to the effects of alcohol [54], they can be reassured that a PP which does not contain wine or any other alcoholic beverage is a healthy dietary option. Lastly, the PP includes all types of meat (white, red, and processed), which is an important source of protein. This is consistent with the emerging evidence of the protective effect of protein intake on frailty [1,7].

This work has several strengths and limitations. Among the former was the prospective design with a sufficient duration to allow for ascertaining a good number of frailty cases. Indeed, there is evidence from clinical trials that the Mediterranean diet lowers the risk of cardiovascular disease in the first few months post-intervention [55,56]; also, previous studies on frailty found a protective effect of the Mediterranean diet after only a few years of follow-up [14,17]. Other strengths were that diet was assessed with a validated instrument and that the results were robust in the sensitivity analyses.

The main limitation was that diet was self-reported. Although we used a validated diet history, we cannot rule out some recall bias. This bias may particularly affect individuals with severe diseases and poor cognition, but the results did not change substantially after excluding these subjects from the analysis. Moreover, recall bias usually leads to underestimation of the study association; nevertheless, it did not impede observation of clear associations between the PP, the WP, and frailty. A further limitation was that factor analysis necessarily involves several arbitrary decisions, including the consolidation of food items into food groups, the number of factors to extract, the method of rotation, and the labeling of the dietary patterns [57]. However, the observed patterns are consistent with those found in studies in other countries [8,31]. Finally, although the analyses accounted for a good number of potential confounders, some residual confounding cannot be ruled out because we only used rather crude measures of depression (diagnosed disease requiring treatment) and cognitive functioning (the Mini-Mental State Examination).

## Conclusions

In this prospective cohort of community-dwelling older adults, two independent dietary patterns derived from consumption data have been shown to predict frailty. Specifically, a PP pattern showed a protective association with frailty while a WP evinced an increased risk of two frailty components: slow walking speed and weight loss. These findings are of practical relevance, first, because they alert us to the fact that the progressive Westernization of the traditional diet in Spain over the last decades [58] may contribute to increased rates, not only of chronic diseases, but also of frailty and its ensuing disability. This may put additional pressure on the already overburdened health and social services. The National Strategy for Nutrition, Physical Activity and Prevention of Obesity in Spain [59] must tackle this issue. Second, they indicate that clinical trials should test whether dietary interventions to adopt a PP and to move away from the WP can be effective in avoiding or delaying frailty.

## Abbreviations

BMI: Body mass index
CI: Confidence interval
IADL: Instrumental activities of daily living
OR: Odds ratio
PP: Prudent dietary pattern
WP: Westernized dietary pattern
